# Special Issue: Discovery of Bioactive Phytochemicals’ Molecular Mechanisms Against Different Diseases Based on Network Pharmacology and Molecular Docking

**DOI:** 10.3390/ijms26104516

**Published:** 2025-05-09

**Authors:** Baojun Xu

**Affiliations:** Food Science and Technology Program, Department of Life Sciences, Beijing Normal-Hong Kong Baptist University, Zhuhai 519087, China; baojunxu@uic.edu.cn

## 1. Introduction

Natural bioactive phytochemicals have shown great potential in disease management [1,2]. With the development of bioinformatics and the deepening of research on active phytochemicals, network pharmacology and molecular docking techniques have been proposed to study the molecular mechanisms of phytochemicals in treating different diseases [3,4,5]. Furthermore, in vivo and in vitro studies can further validate these findings. These studies provide new strategies for disease treatment and management and lay the foundation for developing new natural medicines [6]. With the continuous development and improvement of technology, network pharmacology and molecular docking are playing a greater role in discovering natural bioactive substances, helping to discover more drug therapeutic targets, accelerating the drug development process, and promoting the development of the biopharmaceutical industry [7]. This Special Issue delves into the latest research findings from combining network pharmacology with molecular docking to reveal the molecular mechanisms of active phytochemicals in combating various diseases, revealing recent studies that have attracted widespread attention and made significant discoveries.

## 2. Overview of Published Articles

Bioinformatics analysis is a technical approach that can be combined with network pharmacology and molecular docking, emphasizing the clinical significance of targets in treating related diseases in network pharmacology. Wu’s group revealed the molecular mechanism by which Geniposide inhibits colorectal cancer by regulating oxidative stress using network pharmacology, molecular docking, and bioinformatics [8]. More specifically, the anticancer effect of Geniposide may be achieved through the PI3K–Akt signaling pathway, IL-17 signaling pathway, p53 signaling pathway, NF-κB signaling pathway, and NOD-like receptor signaling pathway. The results of molecular docking, bioinformatics, and network pharmacology screening in this study were consistent, demonstrating their effectiveness.

Although this Special Issue emphasizes the combined use of network pharmacology and molecular docking, both techniques can be used separately for scientific research. Martínez-Alarcón et al. discovered a recombinant Tepary bean (*Phaseolus acutifolius*) lectin (rTBL-1) which can induce apoptosis in colon cancer cell lines [9]. This bioactive effect was related to the differential recognition of β1-6 branched N-glycans and studied using molecular docking. This research has illustrated how the Tepary bean lectin affects the microenvironment within which cancerous cells develop, creating new opportunities to uncover the mechanisms by which this lectin exerts its anticancer effects.

Combining molecular docking and molecular dynamics is also a feasible and efficient research strategy. Alotaiq et al. utilized 3D structure modeling, protein–protein docking, molecular dynamics simulations, and MM/PBSA calculations to assess the binding affinities and functional implications of Lumbricus-derived proteins in relation to SOCS2 activity [10]. This study suggests that targeting the SOCS2 protein may stimulate the biological activity of Lumbricus-derived proteins in the treatment of cardiovascular diseases. Further in vitro and in vivo studies are needed to explore the biological activity of Lumbricus-derived proteins and their potential commercial value.

Yu et al. successfully constructed an engineered *S. cerevisiae* strain (BY-Ro) for ginsenoside Ro biosynthesis using S. cerevisiae BY4741 as the host cell by sequentially transferring these DNA fragments into chemically competent cells of engineering strains and conducting screening and target product detection [11]. Moreover, the ginsenoside Ro is one of the few oleanane-type ginsenosides, which is well known for its unique molecular structure and biological activities.

Ruiz-Alcaraz et al. studied the in vitro anti-inflammatory effect of serious phytochemicals, including phenolic di-caffeoylquinic acid (Di-CQA), flavonoid cyanidin-3,5-diglucoside (Cy3,5DiG), aromatic isothiocyanate sinalbin (SNB), and aliphatic isothiocyanate sulforaphane (SFN), which are sourced from vegetables and fruits [12]. These results indicate that plant compounds from dietary sources can significantly reduce the regulation of the LPS-induced inflammatory factors TNF-α and IL6. This study highlights the potential of plant-derived bioactive compounds as valuable contributors to the prevention or treatment of chronic inflammatory diseases.

Verifying the findings of network pharmacology and molecular docking through in vivo animal experiments is a condensed research strategy. Shu et al. demonstrated the potential of hesperidin to treat CPT-11-induced diarrhea through a combination of network pharmacology, molecular docking, and experimental validation [13]. This study demonstrated that hesperidin significantly alleviated chronic diarrhea in mice and further validated the relevant signaling pathways predicted by network pharmacology. In addition, the interaction between hesperidin and seven key targets regulating cell apoptosis (AKT1, ANXA5, CASP3, HSP90AA1, IGF1, MMP9, and PPARG), as demonstrated by molecular docking, has also been validated through in vivo experiments.

One of the reviews presented in this Special Issue provides a detailed analysis of COVID-19 in children and its relationship with vitamin D, highlighting the impact of pandemic-related confinement on children’s vitamin D levels [14]. It explores how vitamin D acts as a critical regulator of immune responses and its potential role in mitigating the cytokine storm associated with severe COVID-19. The review also evaluates the limited evidence supporting vitamin D supplementation as an adjuvant treatment for COVID-19, emphasizing the need for further large-scale randomized controlled trials to confirm its benefits.

Another review comprehensively examines the therapeutic potential of aminoflavonoids, focusing on their synthesis, biological activities, and the influence of structural modifications on their efficacy [15]. It highlights the anticancer, antimicrobial, antiviral, and anti-inflammatory properties of aminoflavonoids and their metal complexes, emphasizing the importance of the nitrogen atom in enhancing their biological activities. The review also identifies promising candidates like AFP464, which has shown potential in clinical trials, and underscores the need for further research into the mechanisms of action and safety profiles of these compounds to facilitate the development of novel therapeutic agents.

## 3. Conclusions

This Special Issue focuses on the discovery of molecular mechanisms for bioactive phytochemicals against different diseases based on network pharmacology and molecular docking, selecting eight academic papers that delve into the complex molecular mechanisms behind these topics. These papers also highlight the enormous potential for further exploration and discovery in the field of phytomedicine. Looking ahead to the future, the advancement of network pharmacology combined with molecular simulation, bioinformatics, transcriptomics, proteomics, and other related technologies is expected to promote breakthrough research and innovation in the field of phytomedicine.
